# The Role of High‐Frequency Ultrasonography in Managing Adverse Reactions to Exosome‐Based Rejuvenation

**DOI:** 10.1111/jocd.71023

**Published:** 2026-07-01

**Authors:** Yezdan İpek Davarcıoğlu, Ayşenur Botsalı

**Affiliations:** ^1^ Department of Dermatology and Venereology, Gülhane Training and Research Hospital University of Health Sciences Ankara Turkey

To the Editor,

The increasing popularity of exosome‐based therapies in aesthetic medicine is accompanied by a rise in reports of potential side effects, particularly when used off‐label via intradermal injection. Although serious complications such as skin necrosis and persistent granulomatous inflammation after exosome administration have been highlighted in the current literature [1, 2], the challenges in diagnosing these newly emerging safety issues still remain important. We report a case of delayed inflammatory nodules following periorbital exosome injection and discuss the role of high‐frequency ultrasonography (HFUS) as a non‐invasive diagnostic tool in the evaluation and management of these reactions.

A 52‐year‐old woman presented to our clinic with firm, erythematous‐to‐skin‐colored papules along the lateral orbital rims, appearing 2 weeks after a periorbital “rejuvenation” procedure. The patient had received an intradermal injection of a human dermal fibroblast‐derived exosome product, which was originally intended for topical application. The patient reported that post‐procedural papules at the injection sites resolved spontaneously within a few hours. Approximately 2 weeks later, persistent papules developed in the treated areas. Clinical examination revealed multiple non‐fluctuant, non‐tender papules that showed no signs of infection or vascular occlusion (Figure 1A).

**FIGURE 1:**
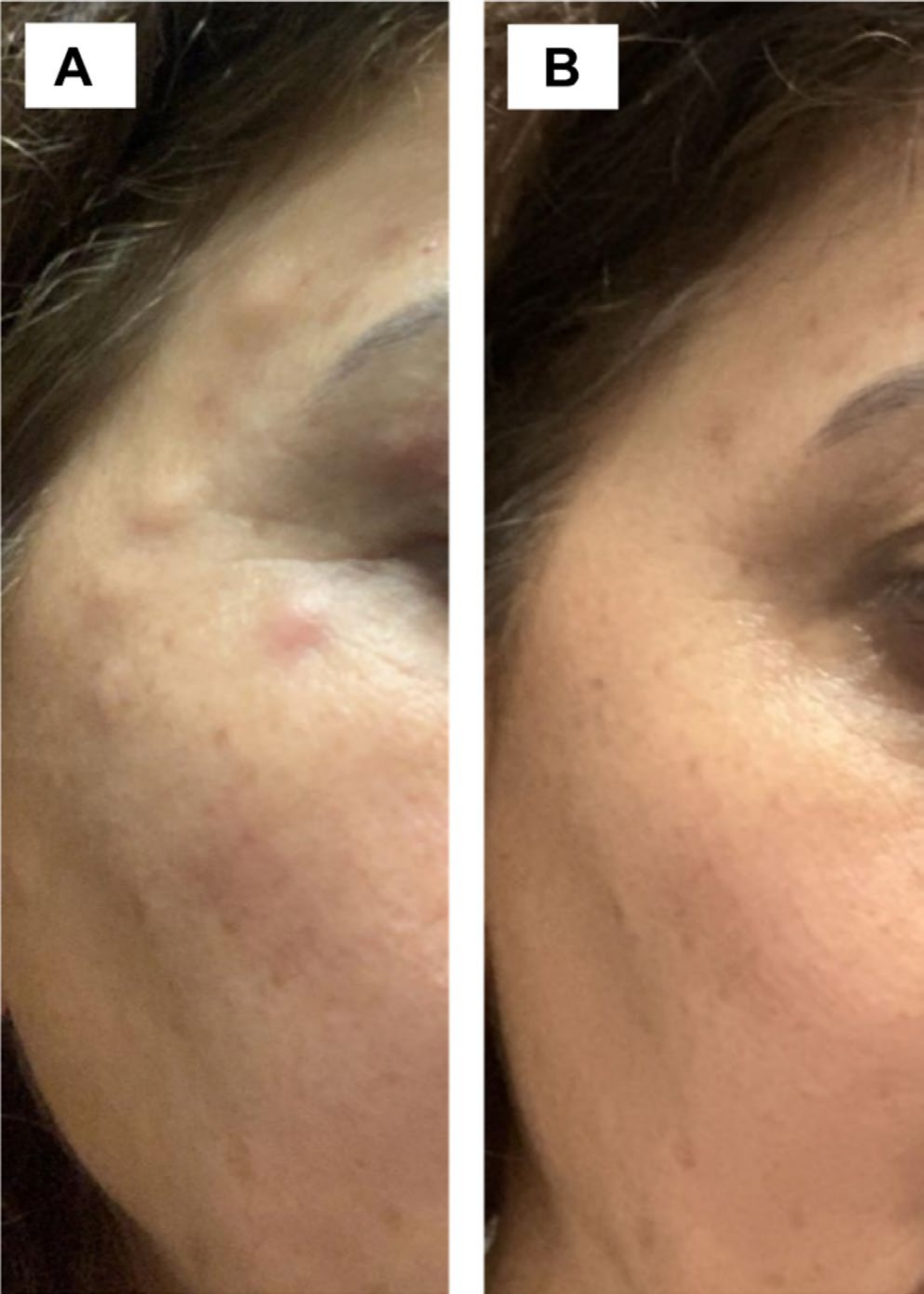
Clinical presentation and treatment outcome. (A) Multiple erythematous‐to‐skin‐colored, firm papules along the lateral orbital rim appearing 2 weeks after exosome injection. (B) Complete resolution of the lesions 1 month after a single intralesional triamcinolone acetonide injection, leaving only mild post‐inflammatory hyperpigmentation.

Given the sensitive periorbital location, the patient declined a skin biopsy. To characterize the lesions, 20‐MHz HFUS was performed, which revealed multiple well‐defined hypoechoic nodules measuring 2–5 mm in diameter at the dermal–subcutaneous interface (Figure 2). No internal vascularity was detected on Doppler examination. These sonographic findings were suggestive of foreign‐body granulomas, consistent with the clinical history of exogenous material injection. Because histopathologic confirmation was not available, the diagnosis remained presumptive and was based on the combined clinical and ultrasonographic findings. Prior to presentation at our clinic, the patient had tried massage, topical corticosteroids, and warm compresses without clinical improvement. Given the persistence of the localized lesions despite these conservative measures, intralesional triamcinolone acetonide injection was selected as a targeted anti‐inflammatory treatment and administered without ultrasound guidance. Triamcinolone acetonide (40 mg/mL) was diluted with 2% prilocaine hydrochloride at a ratio of 0.3–0.7 mL (0.3 mL triamcinolone acetonide and 0.7 mL prilocaine hydrochloride), and approximately 0.1 mL of diluted solution was injected into each nodule. Complete resolution was achieved within 1 month, leaving only mild post‐inflammatory hyperpigmentation (Figure 1B).

**FIGURE 2:**
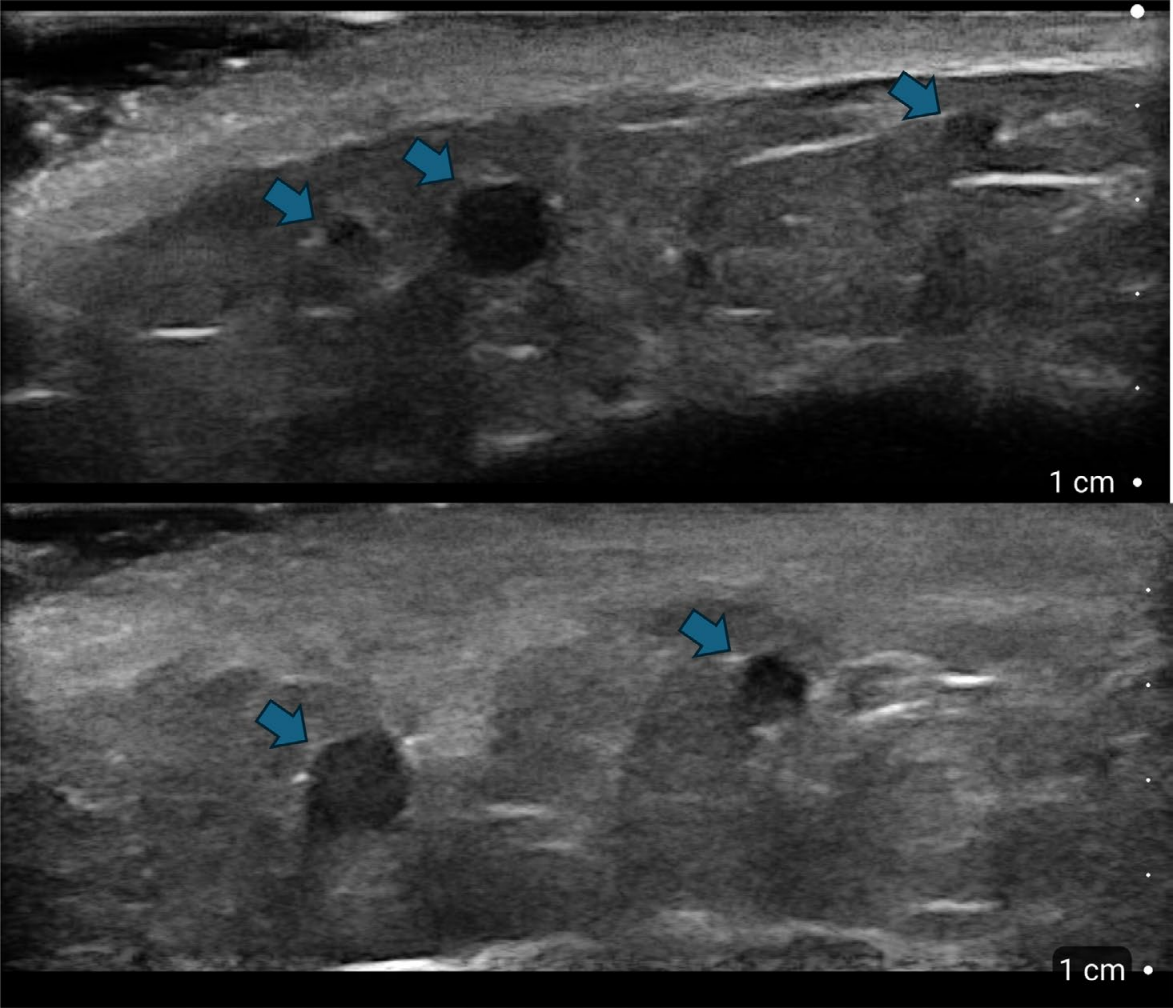
High‐frequency ultrasonography (HFUS) findings. Representative 20‐MHz HFUS image showing well‐defined, avascular, and hypoechoic nodules (blue arrows) at the dermal–subcutaneous interface, highly suggestive of foreign‐body granulomas.

This case supports growing concerns that exosome formulations, especially those not approved for injection, may cause significant inflammatory responses. In our patient, the absence of infectious symptoms and the clear temporal relationship with the injection of a product intended for topical‐use strengthen the suspected causal relationship. Although histopathology remains the gold standard for diagnosing granulomatous reactions, HFUS offers a rapid, real‐time, and non‐invasive alternative, particularly when biopsy is impractical or declined by the patient [3]. HFUS not only determines the morphology and depth of nodules, but also confirms the avascular structure, which is crucial for ruling out vascular complications and planning targeted treatment [4].

As long as exosome‐based products continue to be used off‐label for injectable applications outside of a clear regulatory framework, their safety profiles must be carefully examined. In the United States, the U.S. Food and Drug Administration (FDA) has not approved any exosome‐based products for intradermal injection, and regulatory restrictions in Europe also limit the clinical use of human‐ derived exosomes [5, 6]. As discussions regarding the clinical use of exosomes are still ongoing, documenting even the smallest adverse effects is very important for a more comprehensive understanding of their safety profile. HFUS appears to be a valuable non‐invasive tool in the clinical workflow for managing suspected adverse reactions to these products, providing objective information regarding lesion morphology, depth, and vascularity while facilitating lesion characterization in clinically challenging situations where biopsy is declined or impractical.

## Author Contributions

All authors contributed equally to the conception, data collection, drafting, and critical revision of the manuscript. Both authors have given final approval of the version to be published.

## Funding

The authors have nothing to report.

## Ethics Statement

The authors confirm that the ethical policies of the journal, as noted on the journal's author guidelines page, have been adhered to. As this is a clinical observation report of a single case with standard clinical management, formal IRB approval was not required, but the principles of the Declaration of Helsinki were followed.

## Consent

Written informed consent was obtained from the patient for the publication of this report and any accompanying images.

## Conflicts of Interest

The authors declare no conflicts of interest.

## Data Availability

The data that support the findings of this study are available from the corresponding author upon reasonable request.
